# HIV Surveillance and Research for Migrant Populations: Protocol Integrating Respondent-Driven Sampling, Case Finding, and Medicolegal Services for Venezuelans Living in Colombia

**DOI:** 10.2196/36026

**Published:** 2022-03-08

**Authors:** Andrea L Wirtz, Kathleen R Page, Megan Stevenson, José Rafael Guillén, Jennifer Ortíz, Jhon Jairo López, Jhon Fredy Ramírez, Cindy Quijano, Alejandra Vela, Yessenia Moreno, Francisco Rigual, James Case, Avi J Hakim, Wolfgang Hladik, Paul B Spiegel

**Affiliations:** 1 Center for Public Health and Human Rights Department of Epidemiology Johns Hopkins Bloomberg School of Public Health Baltimore, MD United States; 2 Center for Humanitarian Health Department of International Health Johns Hopkins Bloomberg School of Public Health Baltimore, MD United States; 3 Division of Infectious Diseases Johns Hopkins Bloomberg School of Public Health Baltimore, MD United States; 4 Red Somos Bogotá Colombia; 5 Johns Hopkins School of Nursing Baltimore, MD United States; 6 Centers for Disease Control and Prevention Atlanta, GA United States

**Keywords:** HIV, epidemiology, migrant, Venezuela, Colombia, respondent-driven sampling, case finding, HIV treatment, HIV surveillance, research

## Abstract

**Background:**

Epidemiologic research among migrant populations is limited by logistical, methodological, and ethical challenges, but it is necessary for informing public health and humanitarian programming.

**Objective:**

We describe a methodology to estimate HIV prevalence among Venezuelan migrants in Colombia.

**Methods:**

Respondent-driven sampling, a nonprobability sampling method, was selected for attributes of reaching highly networked populations without sampling frames and analytic methods that permit estimation of population parameters. Respondent-driven sampling was modified to permit electronic referral of peers via SMS text messaging and WhatsApp. Participants complete sociobehavioral surveys and rapid HIV and syphilis screening tests with confirmatory testing. HIV treatment is not available for migrants who have entered Colombia through irregular pathways; thus, medicolegal services integrated into posttest counseling provide staff lawyers and legal assistance to participants diagnosed with HIV or syphilis for sustained access to treatment through the national health system. Case finding is integrated into respondent-driven sampling to allow partner referral. This study is implemented by a local community-based organization providing HIV support services and related legal services for Venezuelans in Colombia.

**Results:**

Data collection was launched in 4 cities in July and August 2021. As of November 2021, 3105 of the target 6100 participants were enrolled, with enrollment expected to end by February/March 2022.

**Conclusions:**

Tailored methods that combine community-led efforts with innovations in sampling and linkage to care can aid in advancing health research for migrant and displaced populations. Worldwide trends in displacement and migration underscore the value of improved methods for translation to humanitarian and public health programming.

**International Registered Report Identifier (IRRID):**

DERR1-10.2196/36026

## Introduction

The economic crisis and political instability in the Bolivarian Republic of Venezuela has led to mass migration in the Americas, displacing approximately 5.4 million Venezuelans as of September 2020, according to the most recent estimates [1]. This humanitarian emergency has been associated with deteriorating health care infrastructure and worsening health outcomes among Venezuelans living in the country, as well as among those displaced to neighboring countries [2]. The re-emergence of previously controlled infectious diseases and resurgence of endemic diseases have been documented and raised concerns of a spillover effect to neighboring countries [2-8]. The COVID-19 pandemic has exacerbated public health concerns and strained the capacity of the receiving countries to meet the health care needs of Venezuelan migrants [9,10].

Gaps in HIV diagnostics and treatment in Venezuela since 2015 have limited the availability of reliable estimates of HIV burden. In 2018, the Pan American Health Organization (PAHO) estimated that 69,308 people living with HIV (PLHIV), 87% of whom were registered to receive antiretroviral therapy (ART), were not receiving them owing to nationwide drug shortages [11]. A coordinated response led by PAHO has improved ART coverage [12], although diagnosis, treatment, and suppression remain suboptimal. The Joint United Nations Program on HIV/AIDS estimates that 100,000 people were living with HIV in Venezuela in 2020, with 71% of PLHIV diagnosed and 55% of those diagnosed receiving ART [13]. No data on virologic suppression rates are available [13]. Less than one-third (30%) of pregnant women living with HIV received ART for prevention of maternal-to-child transmission [13]. Access to HIV treatment for displaced Venezuelans in receiving countries is variable and depends on the national health programs and policies of the host country. Data from other studies show that migrant populations, regardless of the situation or motivation for migration, often face delays to care and have higher risk of AIDS-defining events than nonmigrant populations [14]. Treatment interruptions, including partial or intermittent treatment, can lead to virologic rebound and increase the risk of onward transmission and acquired resistance [2]. Diagnostic delays due to lack of HIV-testing capacity, including in pregnant women, can also lead to ongoing transmission. These concerns, coupled with an estimated 25,000 Venezuelans crossing the Colombian border per day at the peak of the exodus [15,16], underscore the importance of implementing appropriate surveillance methods coupled with access to HIV diagnosis, treatment, and care for migrants.

Colombia currently receives the largest number of displaced Venezuelans in the region. As of February 2021, approximately 1.7 million were living in Colombia [17-19]. Treatment for Venezuelans with irregular migrant status, that is, those who have entered the country outside of official or regular migration channels, is not available through the health system with the exception of prenatal care. Drug donations have made treatment available in Cúcuta, a border city in Colombia, with many Venezuelans living there or crossing the border temporarily to access treatment [20,21]. In other areas of the country, treatment options for migrants with irregular status are limited although several organizations provide HIV testing, support services, and prevention for Venezuelan migrants. Population-based estimates of HIV are absent, but they are needed to inform treatment distribution plans for future drug donations [20] and national health programming. Migrant populations are often excluded or not classified by public health and disease surveillance methods. Traditional epidemiologic surveillance efforts among displaced populations are challenged by lack of sampling frames, mobility, and ethical concerns [22,23]. Migrants do not always reside in well-defined geographic spaces, are frequently dispersed within host communities, and may move multiple times before settling in an area, all of which limits implementation of traditional probability sampling approaches for surveillance. Finally, ethical considerations to protect participants, mitigate stigma, and ensure linkage to HIV care in settings where treatment is not regularly available add further logistical challenges to such surveillance methods [22,23].

This paper describes a protocol for community-led HIV surveillance among Venezuelan migrants residing in Colombia. The protocol expands upon a network-based sampling method by integrating case finding and linkage to care through medicolegal partnerships. The findings aim to inform local treatment distribution plans [20] and country-level and regional HIV programming for migrants.

## Methods

### Design

The BIENVENIR Project (Bienestar de Venezolanos quienes son Inmigrantes y Refugiados) is a cross-sectional design that uses a hybrid sampling and case finding approach, coupled with medicolegal services to link individuals with HIV diagnosis to HIV treatment and care, regardless of migration status. *Red Somos,* a community-based organization, leads this implementation. Staff members are nationals of Venezuela, Colombia, or both, and have expertise in HIV testing, ancillary services, and linkage to care; legal services related to migration; psychology and social work; and community strengthening. This study is conducted among newly arrived Venezuelans living in 4 cities in Colombia. Study findings will generate estimates of HIV prevalence among adults who have arrived in Colombia since 2015, as well as qualitative estimates of engagement along the HIV care continuum among Venezuelan PLHIV. A qualitative, formative research phase was conducted to assess barriers to HIV case and health services in Colombia and to inform the quantitative research methods.

### Formative Research

Key informant interviews with stakeholders (n=29), including humanitarian and health providers, government officials, and medical providers, were conducted in English and Spanish between June and October 2020. In-depth interviews (n=31) and 1 focus group discussion (n=9) with Venezuelans living in Colombia were conducted in Spanish between April 2021 to June 2021. Data collection was conducted remotely by phone or video teleconference to reduce COVID-19 transmission risks. Formative research served to provide contextual information about the humanitarian situation and programming; availability of HIV prevention and care for Venezuelans in Colombia; the impact of COVID-19 pandemic on these issues and research; and to inform decisions related to the incentives, sampling, and development of survey measures. Qualitative findings will also be used to guide subsequent interpretation of surveillance findings. Finally, formative interviews helped to ensure the study is culturally relevant and appropriate.

### Sample and Setting

**Figure 1:**
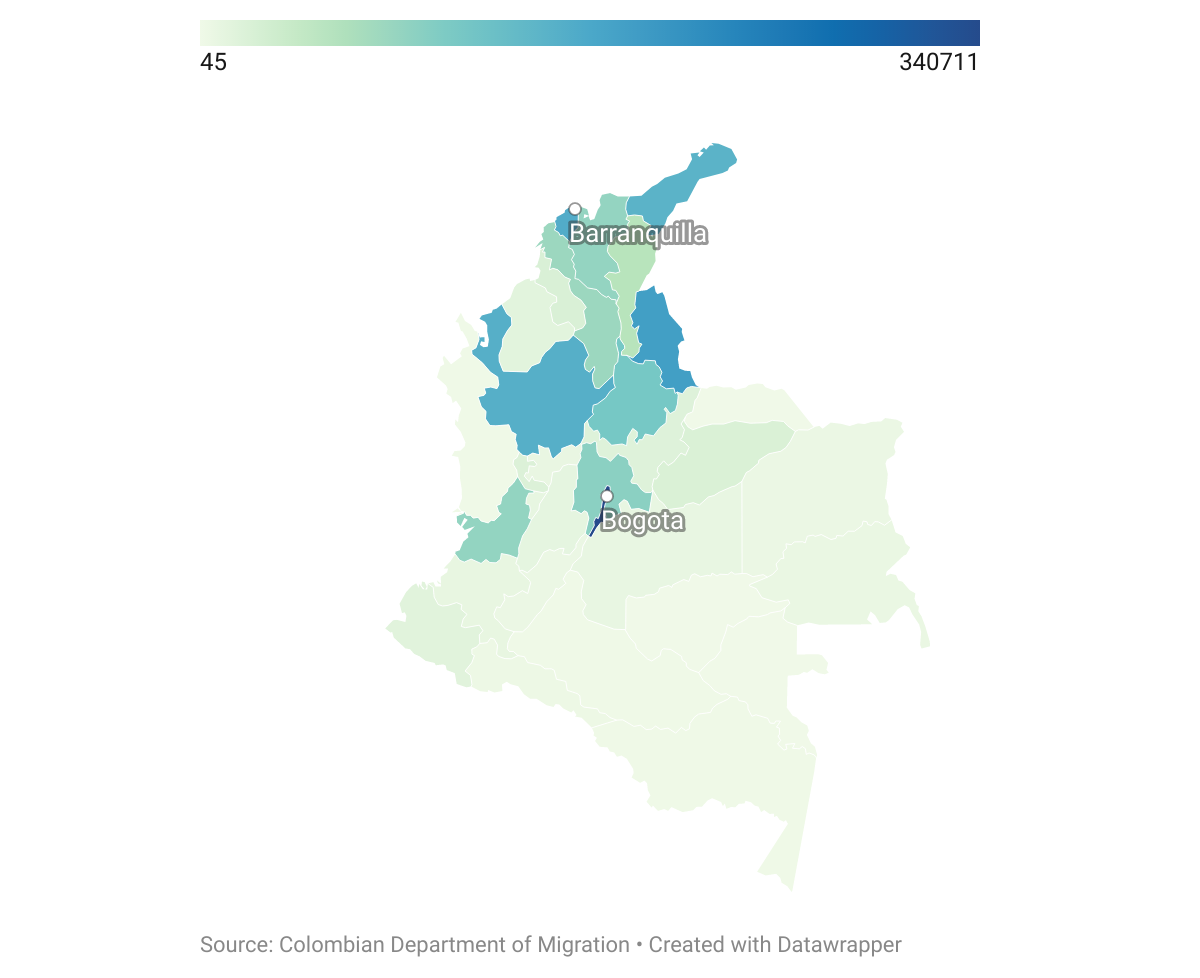
Distribution of Venezuelan migrants in Colombia by department. Source: Colombian Department of Migration, 2021.

Data collection activities are conducted in 2 territories, encompassing the neighboring cities of (1) Bogotá and Soacha and (2) Barranquilla and Soledad (Figure 1) [24]. Locations were selected for the distribution and heterogeneous profiles of Venezuelan migrants, accessibility to humanitarian and health programs, plans for treatment distribution, and lower presence of pendulares (Venezuelans who live in Venezuela but who cross to Colombia regularly to access services) and caminantes (Venezuelans who transit through Colombia to another country). One office was established in each of the 4 cities. All adult Venezuelan nationals (age ≥18 years) who recently migrated to Colombia are eligible to participate. To ensure recruitment depth in the network of Venezuelans, only 1 member in an immediate family is eligible to participate. Inclusion criteria are as follows: Venezuelan national based on self-report (proof/documentation of nationality is not requested), born in Venezuela based on self-report, age ≥18 years, migrated to Colombia as of 2015 or later, currently residing (ie, spends most of their nights) in the study city, and has a valid study coupon to enrollment (except seeds). Participants with any of the following characteristics are excluded from participation: previous participation, have an immediate family member in the same household who participated, currently resides outside of Colombia, reports being in transit through Colombia (ie, reports an immediate destination outside of Colombia), or lacks capacity to consent. Enrolled participants are asked to provide their names and mobile phone or WhatsApp numbers for recontact and for identification of duplicate participants.

### Sampling

Respondent-driven sampling (RDS), a chain referral sampling method that employs limited referrals within peer networks to achieve target sample sizes, is used to accrue the study sample. RDS is widely used across international settings to sample populations that lack a sampling frame. Research has shown that with sufficient recruitment depth, biases associated with initial peer referrals are minimized [25-27], and there is an increased ability to identify previously undiagnosed HIV infections and PLHIV who have fallen out of care [27,28]. RDS thus provides an opportunity to generate unbiased estimates as well as to link surveillance with HIV clinical services. Estimation methods generate survey weights based on participant network size, and network features have been developed to calculate population-based prevalence estimates [25]. RDS has been previously used among migrants and displaced populations to a limited degree in international settings [29-33] as well as among key populations affected by HIV in Colombia [34-36].

Sampling commenced at the end of July and August 2021 in Bogotá/Soacha and Barranquilla/Soledad, respectively, and is ongoing. Recruitment started with 19 “seeds” (9-10 per territory)—well-networked individuals who were selected from the target population. Seeds were purposively selected on the basis of being well-respected and influential among peers, socially networked (know at least 10 Venezuelans outside of their household), and diverse in characteristics (eg, age, gender, geographic residence within each city). To minimize cluster effects, we identified and enrolled seeds who did not know each other and who likely did not have overlapping networks. Additional seeds may be initiated at a later date if prior seeds fail to produce peer referrals or if recruitment slows.

Seeds participate in all study activities and are asked to invite 4 adult Venezuelan peers (recruits) to participate in study activities, which is the first sampling wave. Eligible and participating recruits are then asked to refer up to 4 more peer Venezuelans. At the end of each study visit, participants undergo a brief training on how to distribute coupons and refer peers to the study. Participants have the option to use paper or electronic coupons via SMS text messages or WhatsApp to refer peers. Coupons contain study contact information and unique codes that anonymously link seeds/recruiters to recruits for analysis. Although documentation of regular migration status is necessary to acquire a phone in Colombia, 70% of Venezuelan migrants in Colombia report using mobile phones [37], and anecdotal reports support the use of mobile technology. Participants receive automated SMS text messages or WhatsApp notifications to remind them to distribute coupons or to notify them when coupons have been used and they can retrieve their incentive. As is typical in RDS research, participants are provided with a secondary incentive (COP 10,000 or USD 2.60) for each eligible and participating referred peer. Participants return within days to weeks to obtain their secondary incentive and are asked at that time to complete a brief survey about their experience of referring peers and potential biases in the referral process.

RDS is monitored in real time by using the RDS-Analyst platform [38] to ensure that sampling has reached appropriate recruitment depth (waves) to provide unbiased population-based estimates. The data management team monitors for convergence, bottlenecks, homophily, and population proportions for key indicators, including HIV infection and socially salient variables [25]. Monitoring is also used to identify whether any RDS networks may oversample certain key populations. Chains that continue to sample the same group (including men who have sex with men, people who inject drugs or engaged in sex work, and transgender populations) for more than 2 waves without the entry of other populations will be stopped. We aim to achieve a recruitment depth of at least 8-10 waves to minimize bias associated with the initial seed selection.

### Data Collection

Participation consists of a sociobehavioral survey and dual rapid testing for HIV and syphilis infection, followed by confirmatory testing. At entry, participants undergo screening for eligibility and written consent procedures in a private office space. Participants completed a literacy screener using the Spanish language version of the Rapid Estimate of Adult Literacy in Medicine–Short Form [39] prior to completing a self-administered electronic survey questionnaire or an interviewer-administered option depending on the literacy score. Participants with less frequent technology use can also request an interviewer-administered survey. Following best practices in survey research, we used a mode-enhancement construction, which develops the data collection instruments to be optimized for the main mode (here, self-administered surveys given high literacy) with the auxiliary mode (interviewer-administered) designed to be equivalent [40,41]. Participants who complete the survey and dual rapid test are provided an incentive of COP 30,000 (USD 7.85).

We use a secure system for managing participant tracking and data within the study. This system was developed internally for observational (including RDS) and clinical trial research and was customized to this study [42,43]. The system is a web application and accessible in English and Spanish languages. User access to the system is location- and role-based and protected with a username and password. Entered data are encrypted in transit and stored in a secure server at Johns Hopkins. Study staff can register a participant and follow the status of the participant with respect to the completion of study activities, generate RDS coupons, send notifications, and track receipt of primary and secondary incentives. The system has built-in algorithms to check for duplicate participant registrations and to validate returned coupons for eligibility. All exported data are automatically stripped of identifiers and are linked to a participant via a unique study identifier.

### Survey Measures

Survey measures included individual, social, and structural domains, drawing upon previously developed measures, as applicable (Table 1) [26,44-58]. Other health indicators beyond HIV prevention and care measures are included for assessing overall health status, identifying other health concerns that may particularly affect PLHIV (eg, malnutrition), and identifying correlates of HIV infection. Inclusion of other health measures also helps to minimize the stigma associated with an HIV survey questionnaire. All study measures and participant materials are translated into Spanish and back translated for quality.

**Table 1:** Domains and measures included in the survey questionnaire.

Domain	Measures
Demographics	Basic demographics adapted from the Colombia Demographic Health Survey [52] Food (in)security as measured by the US Food and Drug Administration food security scale [44]
Migration and displacement	Displacement history (timing, location of residence in Colombia, and migration status, eg, regular or irregular)
Health	Recent health history Self-rated health [53] Body mass index (self-reported height and weight) Depression symptoms measured by the Patient Health Questionnaire for Depression and Anxiety [54,55] Alcohol measured by Alcohol Use Disorders Identification Test-Concise [49,50] and drug use [56] COVID-19 symptoms and testing history
HIV: behavioral risks and uptake of HIV prevention and care	HIV acquisition risk behaviors adapted from World Health Organization biobehavioral survey guidelines for populations at risk for HIV [56] Access to and engagement in HIV services: HIV testing, HIV prevention [56] HIV care continuum: self-reported diagnosis of HIV, engagement in HIV care including CD4 testing, viral load testing, and suppression [45,46,57], including country(ies) where care and treatment were accessed Access to, uptake, and adherence to HIV treatment adapted from the Adult AIDS Clinical Trials Group survey measures [58]
Social measures	Discrimination using the Everyday Discrimination Scale (short version) [51] Violence victimization using the Assessment Screen to Identify Survivors Toolkit for Gender-Based Violence screen for displaced populations [47,48]
Respondent-driven sampling	Social network size questions used for respondent-driven sampling weighting procedures [26]

### Biological Measures

Biological measures include rapid HIV and syphilis screening using Standard Diagnostics BIOLINE HIV/Syphilis Duo with finger-prick blood specimens. Standard Diagnostics BIOLINE HIV/Syphilis Duo has a reported sensitivity of 99.8% and specificity of 100% for anti-HIV antibody detection and a reported sensitivity of 90% and specificity of 99.9% for anti–*Treponema pallidum* antibody detection [59]. Screening results are available within 20 minutes and provided to the study participants during the study visit. Participants with a reactive result on either or both tests are asked to provide an additional venous specimen for laboratory-based confirmatory testing. Specimens are transported the same day to the local reference laboratory. Confirmatory testing for participants with a positive HIV screen follows national testing algorithms and policies [60] and is conducted using the MP Bio HIV BLOT 2.2, a qualitative enzyme immunoassay for antibody detection of HIV-1 and HIV-2. For clinical purposes, and in keeping with national guidelines [60], CD3, CD4, CD5, CD8, and viral load tests are also performed at the same time by the laboratory. HUMAN Diagnostics Syphilis Rapid Plasma Reagin Test was used for confirmatory testing and identification of active syphilis for participants with positive syphilis screen. All laboratory results are available to the study team within 2 weeks; negative results are communicated by phone to the participants within 1 business day of receipt. Participants with confirmed HIV, syphilis, or both are contacted within 1 business day to arrange a time to deliver the results in person. All laboratory results are provided to the participant. The medicolegal triage process begins immediately at that time. Laboratory results are shared with medical providers upon participant request to assist with treatment decisions that are made by the patient and provider.

### Medicolegal Services

Lawyers employed by *Red Somos* support the legal process of registering Venezuelans with irregular migration status to support access to care through the national program. All participants identified to be living with HIV (previously or newly diagnosed) or who have active syphilis will undergo a legal triage in which their legal status in Colombia will be reviewed by the assigned lawyer. For those with irregular migration status, staff lawyers initiate and support the completion of necessary paperwork and processes to acquire *permiso especial de permanencia* (or permit of stay), a *salvoconducto* (a paper demonstrating the regularization process has been initiated), or the forthcoming *estatuto temporal de protección* (or temporary statute of protection) documentation*.* Access to medical care and treatment under the national health system generally requires possession of one of these forms of documentation or other documents in special circumstances.

The process to obtain a *salvoconducto* takes 15 days maximum, while the process to access ART through the national health system (for any individual, inclusive of Colombian citizens) takes up to 30 days. During the period, while participants wait for their permit of stay or *salvoconducto* request to be processed, *Red Somos* will link the participants to local clinics and agencies that currently provide interim HIV care and treatment to the Venezuelan population who they regularly serve. At this point, the providers and patients will make treatment decisions with consideration of viral load, CD4 counts, and any critical comorbid conditions. Those agencies can initiate stopgap ART in as little as 8 days.

### Case Finding

To support efforts to identify new or undiagnosed infections, we employed a hybrid RDS–case finding approach. Case finding follows World Health Organization and Centers for Disease Control and Prevention guidelines for partner notification services and were adapted to reflect community recommendations to mitigate risk of violations of privacy, breaches in confidentiality, and coercive medical practices [61-63]. Participants with laboratory-confirmed HIV are invited to participate in partner notifications services to identify and support HIV and syphilis testing of sexual or injecting partners. Although encouraged to invite contacts to get tested, participants have the option to decline or to request anonymous partner notification through study staff. Case finding is open to all adult partners (age ≥18 years) of participants with laboratory-confirmed HIV, regardless of country of origin or citizenship status. Children are not eligible, but clinical referrals are offered to parents of children who may have been infected perinatally. During posttest counseling and linkage to care, participants who opt-in to case finding activities are trained on how to disclose their HIV status and invite partners to participate in HIV and syphilis testing. Participants are provided with a uniquely identified coupon that contains study contact information, which enables anonymous linkage of participants and contacts. The case-finding coupon is distinct in appearance from the RDS coupon but functions in a similar way. Any persons found to be living with HIV or syphilis through the case finding are connected to care through the same pathways. Brief service-focused interview questionnaires are administered among case-finding contacts to identify transmission risk behaviors and access/use of HIV prevention and testing.

### Sample Size

Assuming a 1% HIV prevalence among general population, based on reports from local providers that suggest a range of 0.5% prevalence among adults to 1.5% prevalence in antenatal care surveillance, alpha .05, 0.005 margin of error, and design effect of 2 that has been suggested for RDS [64-66], we estimated that a sample size of 3043 per territory (approximately 6100 overall) is needed to estimate population HIV prevalence. This sample size provides a sufficiently small sampling fraction required by most of the RDS estimators [25], given that the Venezuelan migrant populations are estimated to exceed 115,000 persons in both territories. Individuals who are enrolled in case finding activities will contribute to the total enrolled sample. Assuming an HIV prevalence as high as 1.5% and an average of 2 contacts (case finding referrals) per index participant, we estimate that an additional 190 individuals will be enrolled in case finding (95 per territory) for HIV. Assuming 3% prevalence of syphilis, an additional 360 case finding participants will be enrolled for syphilis.

### Analytic Plan

Basic descriptive analysis will be performed to estimate the prevalence of key demographic and health characteristics of the sample population. Primary analysis will focus on estimation of HIV prevalence among the general population of Venezuelans residing in the 2 territories, with estimates separately for each territory. Among participants living with HIV infection (prevalent or new diagnoses), we will assess engagement in the HIV care continuum, including the proportion who report being aware of their infection, engaged in HIV care, currently on ART, completing viral load testing in the last 6 months, and having suppressed viral load [67]. Subgroup analyses will be conducted to estimate HIV prevalence by HIV risk behaviors, gender, and age. All descriptive analyses will include unweighted and RDS-weighted population estimates for the adult Venezuelan population [68]. RDS-weighted analysis will be performed using Stata (StataCorp LLC) [69] or RDS-A (RDS-Analyst) [38] software and selecting the estimator most appropriate based on the sample characteristics. Analyses will incorporate RDS survey weights based on self-reported network size to calculate population prevalence. Bootstrapping procedures will be performed to calculate associated 95% confidence intervals [69]. Descriptive analysis will be conducted among case-finding participants to assess the demographic and social characteristics of contacts. Except for case-finding participants who are recruited via RDS or otherwise eligible for RDS, unweighted estimates will be calculated given that they are not part of the original RDS network chains. Data will be combined with RDS participant data to calculate unweighted HIV and syphilis positivity estimates.

### Ethical Considerations and Participant Protection

Study activities were reviewed and approved by the ethical review committee at the Universidad el Bosque in Bogotá, Colombia, and the Institutional Review Board at Johns Hopkins School of Public Health (28223). The protocol was also reviewed in accordance with Centers for Disease Control and Prevention human research protection procedures. Formative research with stakeholders was deemed not human subjects research and commenced prior to other study activities.

This study uses multiple strategies to address unique social risks that underlie research with migrant populations, which go beyond risks typically associated with HIV surveillance. Risks for migrant populations largely encompass concerns for social harms related to stigma and discrimination as well as barriers to access to services, particularly for those with irregular migration status. First, we use a vague study title, *BIENVENIR,* to avoid perceptions of increased risk of HIV among migrants, should others learn about the study. No information that would identify the study focus on HIV or among migrant populations is included in recruitment materials or other outward facing materials. Prior to the implementation of study activities, appropriate referral pathways for HIV, syphilis, ancillary health, and humanitarian services (eg, nutrition, housing, mental health, social support) were identified for participant referral. Our electronic survey is programmed to flag to staff instances when a participant self-reports symptoms of depression or anxiety, food insecurity, hazardous alcohol use, and violence victimization. Although all participants are offered locally tailored resource guides, these individuals will be provided with a more in-depth discussion about resources and services specific to their needs and living situation.

The onset of the COVID-19 pandemic occurred between the funding of this project and the initiation of the study activities. Study launch was delayed during the early peaks of the pandemic, and formative research was conducted through secure remote methods at that time. A separate and extensive COVID-19 biosecurity protocol was developed for in-person data collection, and it aligns with local policies. The biosecurity protocol was submitted to all ethical and protocol review committees, and it underwent additional review and approval by an independent Human Subjects Research Restart Committee at Johns Hopkins University before in-person research commenced.

## Results

**Figure 2:**
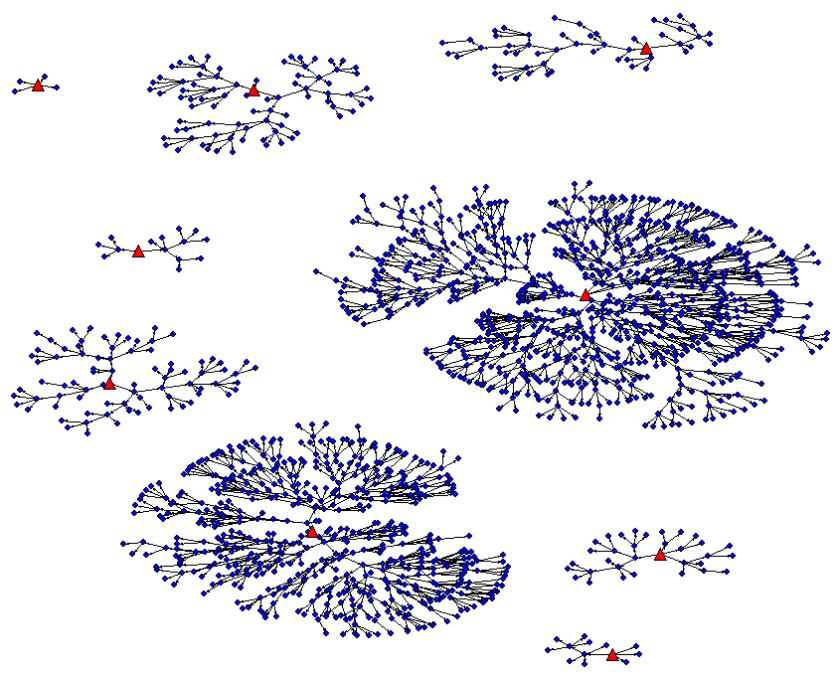
Respondent-driven sampling network graphs of participants in Bogotá and Soacha. The large red triangular nodes represent seeds, and the small blue circular nodes represent recruits.

**Figure 3:**
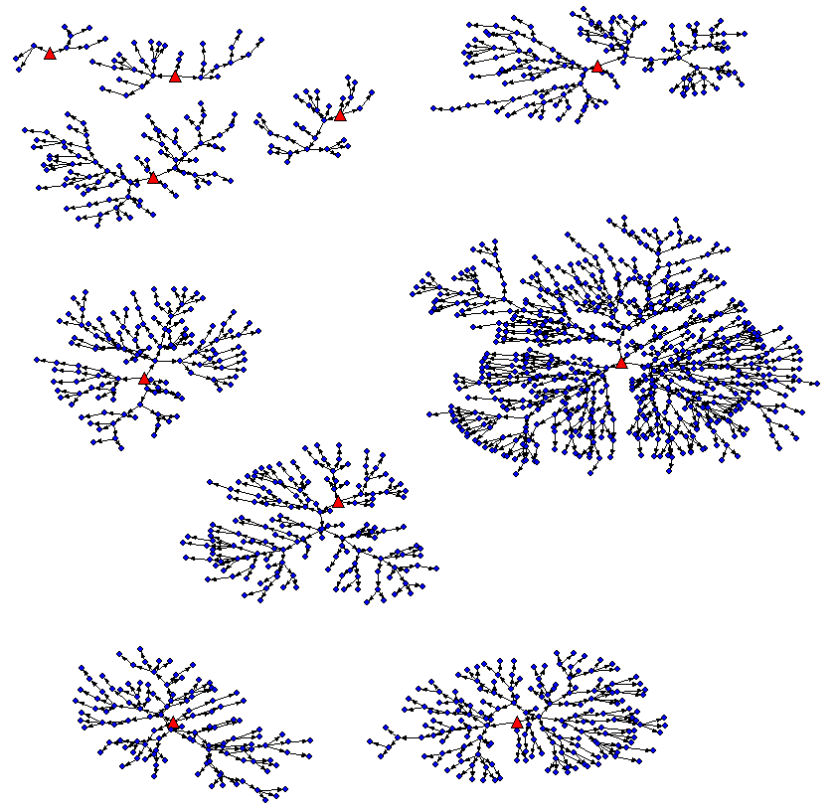
Respondent-driven sampling network graphs of participants in Barranquilla and Soledad. The large red triangular nodes represent seeds, and the small blue circular nodes represent recruits.

As of November 8, 2021, 3278 people have been screened and 3105 participants have been enrolled across sites, inclusive of 20 seeds, and we have reached a maximum recruitment depth of 12 waves thus far (Figures 2 and 3). The enrollment is expected to end by February/March 2022. The number of new participant screenings range from 20-27 per weekday in each territory; Saturday data collection sessions experience higher no-show rates with 9-20 screened per day. Table 2 displays the characteristics of the participants enrolled to date.

**Table 2:** Demographic and other characteristics of the study participants as of November 8, 2021.

Characteristics				Territory				
				Bogotá and Soacha (n=1684)		Barranquilla and Soledad (n=1421)		Total (N=3105)
**Demographics**								
	Age (years), median (IQR)		32 (26-41)		33 (26-41)		32 (26-41)	
	**City of residence, n (%)**							
		Bogotá	861 (51.1)		0 (0)		861 (27.7)	
		Soacha	822 (48.8)		0 (0)		824 (26.5)	
		Barranquilla	0 (0)		861 (60.6)		861 (27.7)	
		Soledad	0 (0)		560 (39.4)		558 (18)	
	**Migration status, n (%)**							
		Regular	522 (31)		250 (17.6)		772 (24.9)	
		Irregular	1162 (69)		1171 (82.4)		2333 (75.1)	
	**Gender identity, n (%)**							
		Male	562 (33.5)		351 (24.7)		913 (29.5)	
		Female	1091 (65.1)		1041 (73.3)		2132 (68.8)	
		Transgender or Nonbinary	24 (1.4)		29 (2)		53 (1.7)	
	High literacy (Rapid Estimate of Adult Literacy in Medicine–Short Form>6, reference<6), n (%)		1485 (89.4)		889 (63)		2374 (77.3)	
**HIV behavioral risks and testing history, n (%)**								
	Lifetime injecting drug use (reference: no)		37 (2.2)		22 (1.5)		59 (1.9)	
	Reports sex with a cis man (reference: no; denominator cis men and trans women, n=941)		53 (9.3)		27 (7.3)		80 (8.5)	
	Sex work (last 12 months)		32 (1.9)		30 (2.1)		62 (2)	
	Lifetime HIV test (reference: no)		1027 (61.2)		681 (48)		1708 (55.1)	
	Past diagnosis of HIV (reference: last test negative or unknown; n=3099)		7 (0.4)		9 (0.6)		16 (0.5)	

## Discussion

Early evaluation of enrollment and participant data show early signals that the methods described here are both feasible and acceptable for research in this context. The hybrid RDS–case finding approach is an innovation in RDS research, with the goal of increasing our ability to identify new or undiagnosed infections among partners and providing linkage to care. Modification of RDS to permit electronic referral of peers via SMS text messages and WhatsApp enables safe referral of peers while maintaining social distancing in the context of COVID-19. Use of text-message referrals builds on common communication pathways and appears to efficiently support peer referral. Given the tenuous access to HIV treatment of Venezuelans in Colombia with irregular migration status, the integration of medicolegal services in posttest counseling aims to increase access to HIV care, decrease time to ART initiation, as well as reduce untreated syphilis. The high recruitment rate thus far is a testament to the efficiencies of RDS and to the model of community-led research implementation and comprehensive service provision inclusive of HIV prevention and linkage to care, legal services, and other ancillary services. These findings will have direct relevance to Colombia, but methods and lessons learned from this study can be adapted for use across diverse settings with numerous health outcomes. With almost 272 million international migrants globally and over 82 million forcibly displaced persons, of whom 55 million are internally displaced due to conflict and insecurity as of 2020 [70,71], such methods are increasingly valuable for understanding and informing strategies related to migrant, humanitarian, and public health.

## Abbreviations

ART: antiretroviral therapy
BIENVENIR: Bienestar de Venezolanos quienes son Inmigrantes y Refugiados
PAHO: Pan American Health Organization
PLHIV: people living with HIV
RDS: respondent-driven sampling
